# Glomerular Crescents in Adult Lupus Nephritis: Clinical and Pathological Insights From Saudi Arabia

**DOI:** 10.1155/tswj/1894247

**Published:** 2026-07-29

**Authors:** Salem H. Al-Qurashi, Muhammad Abdul Mabood Khalil, Hinda Hassan Khideer Mahmood, Fadel A. Alrowaie, Abdullah Mohammed Almansour, Rayan Mohammed H. Alghamdi, Lama Saleh Alghamdi, Maram Majid Alsharif, Rawan Ahmed Alghamdi, Ammar Elgadi, Nihal Mohammed Sadagah

**Affiliations:** ^1^ Renal Diseases and Transplantation Centre, King Fahad Armed Forces Hospital, Jeddah, Saudi Arabia, kfafh.org; ^2^ Department of Medicine, Section of Nephrology, King Fahad Medical City, Riyadh, Saudi Arabia, kfmc.med.sa; ^3^ Department of Medicine, King Fahad Armed Forces Hospital, Jeddah, Saudi Arabia, kfafh.org; ^4^ College of Computing, Department of Computer and Artificial Intelligence, Umm Al-Qura University, Makkah, Saudi Arabia, uqu.edu.sa; ^5^ Faculty of Medicine, University of Khartoum, Khartoum, Sudan, uofk.edu

**Keywords:** crescent burden, crescent formation, lupus nephritis, prognosis, proliferative lupus nephritis, renal outcomes

## Abstract

**Background:**

Crescent formation is a marker of severe kidney injury in lupus nephritis (LN), but data from Saudi Arabia are scarce. We studied how crescents affect clinical features, pathology, and outcomes in Saudi patients.

**Methods:**

We reviewed 100 adults with biopsy‐proven LN (2003–2024), classifying them by the presence and type of crescents (cellular, fibrocellular, fibrous). Crescent burden was calculated as the percentage of glomeruli involved; ≥ 7.39% was considered high. The composite renal outcome included estimated glomerular filtration rate (eGFR) < 20 mL/min/1.73 m^2^, ≥ 20% decline in eGFR, doubling of serum creatinine, or dialysis or transplant. Outcomes were analyzed using Kaplan–Meier curves and multivariable Cox regression.

**Results:**

Out of 100 patients with LN, 41 (41%) had crescents. Those with crescents had lower eGFR (71.41 ± 43.92 vs. 100.12 ± 45.63 mL/min/1.73 m^2^, *p* = 0.002), lower albumin (25.51 ± 6.17 vs. 29.81 ± 7.56 g/L, *p* = 0.004), and higher activity scores (7.93 ± 3.05 vs. 2.27 ± 2.33, *p* < 0.001) than non‐crescent patients; chronicity scores were similar. Kaplan–Meier curves suggested that patients with high crescent burden (≥ 7.39%) had worse event‐free survival, but crescent burden was not an independent predictor. Instead, older age, higher baseline creatinine, elevated CRP, and fibrous crescents were linked to worse outcomes. Treatment response did not differ by crescent burden (*p* = 0.294).

**Conclusion:**

Crescents indicate more active disease and worse kidney function at diagnosis. While a high crescent burden may signal poorer short‐term outcomes, long‐term prognosis depends more on other clinical and pathological factors. Assessing both clinical presentation and biopsy findings is important for managing LN.

## 1. Introduction

Crescent formation reflects a severe form of glomerular injury driven by intense immune‐mediated inflammation. It develops when damage to the glomerular capillary wall allows fibrin and inflammatory products to enter Bowman’s space. This leads to the proliferation of parietal epithelial cells and accumulation of macrophages [1]. Crescents are made up of cells, fibrin, and fibrous tissue [1]. If more than 75% of the crescent is cells and fibrin, it is called a cellular crescent. Crescents with 25%–75% cells and fibrin are considered fibrocellular. When over 75% of the crescent is fibrous tissue, it is classified as a fibrous crescent [1]. In the context of lupus nephritis (LN), crescent formation is most commonly observed in proliferative forms of the disease (Class III and Class IV) as defined by the International Society of Nephrology/Renal Pathology Society classification (ISN/RPS) classification [2]. Although crescents are incorporated into the activity index as a marker of active inflammation, they are not used independently to define LN class [3]. Instead, they reflect severe glomerular injury, accompanied by other features such as endocapillary hypercellularity and necrosis. Therefore, their clinical significance should be interpreted within the broader histopathological context of LN rather than as isolated findings. Crescent formation has been reported in approximately 50% of patients with proliferative LN [4]. Their presence has generally been associated with more severe clinical presentation and poorer renal outcomes, although the prognostic impact of crescent burden and subtype remains variable across studies.

Clinically, crescentic LN is often associated with impaired renal function [4]. Histologic activity scores are usually higher at diagnosis [5]. However, the impact of crescents on prognosis is variable. It may depend on the number of crescents and their composition. Cellular crescents often reflect active, potentially reversible inflammation, whereas fibrous crescents represent established irreversible injury [1]. Emerging data suggest that overall renal outcomes may be more closely driven by associated clinical factors and concurrent histologic activity than by crescent formation alone [4, 6].

Crescents are known to be linked with severe LN [4], but data from Saudi Arabia and the Middle East are limited. Most studies on crescentic LN have been conducted in Western and East Asian populations, so regional data are scarce [7]. Few studies have examined crescents in detail or assessed how the proportion of affected glomeruli influences outcomes [8–10] in Saudi Arabia and middle east countries. Disease patterns, immune characteristics, and treatment practices differ across regions. As a result, the clinical impact of crescents in Saudi patients remains poorly defined. The main aim of this study was to assess the impact of crescent presence and extent on renal outcomes in Saudi patients with LN. The secondary aims were to compare clinical and histopathological features between patients with and without crescents, to evaluate whether crescent type (cellular, fibrocellular, or fibrous) affects outcomes, and to identify factors associated with a poorer renal prognosis in patients with crescentic disease.

## 2. Methods

This retrospective observational study was conducted at King Fahad Armed Forces Hospital in Jeddah and King Fahad Medical City in Riyadh, Saudi Arabia. The main aim of this study was to assess the effects of crescent presence and extent on kidney outcomes in Saudi patients with LN. Secondary aims were to compare clinical and pathological features between patients with and without crescents, to examine whether crescent type (cellular, fibrocellular, or fibrous) influences outcomes, and to identify factors associated with a worse renal prognosis in patients with crescentic disease. The study was approved by the Hospital Research Ethics Review Committee, conducted in accordance with the Declaration of Helsinki, and patient confidentiality was maintained throughout.

Adults with biopsy‐proven LN from 2003–2024 with complete baseline clinical and laboratory data who received standard induction and maintenance therapy were included. Pediatric patients were excluded because the study was conducted in an adult nephrology unit. Management protocols, disease progression, and follow‐up strategies differ significantly between pediatric and adult populations with LN. To ensure a homogeneous cohort and maintain consistency in clinical assessment and treatment pathways, only adult patients were included. Patients were excluded if they had other kidney diseases, incomplete records, prior kidney transplants, were on chronic dialysis before biopsy, were under 18 years of age, or were lost to follow‐up before renal outcomes could be assessed. Renal biopsy specimens were considered adequate for inclusion if they contained at least 10 glomeruli. A total of 100 patients satisfying the selection criteria were included. Ten patients were excluded due to insufficient tissue. No formal sample size calculation was performed, and all eligible cases during the study period were included. All available clinical, laboratory, and histopathological variables were extracted from medical records and pathology reports. Missing data were not imputed, and analyses were performed using available case data.

Patients were divided into two groups based on the presence of crescents in kidney tissue: cellular, fibrocellular, or fibrous. The non‐crescent group had no crescents, while the crescent group included any type of crescent.

All kidney biopsy specimens were processed using standard light microscopy, immunofluorescence, and, in a selected group, electron microscopy (EM). EM was performed in selected cases depending on institutional availability at the time of biopsy processing. No predefined selection criteria were applied, and all cases (70) with available EM data were included in the study. All biopsies were stained with hematoxylin and eosin (HE), periodic acid–Schiff (PAS), and Masson trichrome. Two pathologists independently reviewed and reclassified the biopsies according to the ISN/RPS 2018 criteria, blinded to clinical data, and reached consensus in cases of disagreement [11]. Renal tissue activity and chronicity were scored using both the old [11, 12] and the modified National Institute of Health (NIH) LN indices, as revised by the ISN/RPS 2018 classification [3]. The activity index reflects ongoing, potentially reversible inflammatory lesions, such as endocapillary hypercellularity, cellular crescents, and necrosis, whereas the chronicity index reflects irreversible damage, including glomerulosclerosis, fibrous crescents, and interstitial fibrosis/tubular atrophy. In cases with isolated focal crescents in class I/II LN, classification was based on the dominant histopathological pattern according to ISN/RPS criteria. These cases were re‐reviewed by the renal pathologist to confirm the final classification. Immunofluorescence microscopy was performed to assess immune complex deposition. The “full house” pattern was defined as positive staining for IgG, IgA, IgM, C3, and C1q, whereas “partial house” referred to incomplete staining with the absence of one or more of these immunoreactants in LN biopsies.

The proportion of crescents for each patient was calculated as the number of glomeruli containing crescents divided by the total number of glomeruli in the biopsy, multiplied by 100. A crescent extent cutoff of 7.39% was used to define higher versus lower crescent burden, based on a previously published large cohort study by Tao et al., which evaluated crescent percentage as a prognostic marker in LN using the ISN/RPS 2018 classification system [13].

Composite renal outcome was defined as ≥ 1 of eGFR < 20 mL/min/1.73m^2^, ≥ 20% decline in eGFR, doubling of serum creatinine, need for dialysis, or transplant. A complete response was described as a urine protein–creatinine ratio < 0.5 g/g, with stabilization or improvement in kidney function (within ±10%–15% of baseline), achieved within 6–12 months without rescue therapy. Partial response was defined as ≥ 50% reduction in proteinuria to < 3 g/g, with stabilization or improvement in kidney function within ±10%–15% of baseline [14].

Clinical, laboratory, treatment, and histopathologic variables were obtained from medical records and renal biopsy reports. Clinical variables included age at diagnosis, sex, blood pressure, presence of hypertension or diabetes mellitus, and duration of follow‐up. Baseline laboratory parameters comprised serum creatinine, eGFR, 24‐h urine protein excretion, serum albumin, hemoglobin, white blood cell and platelet counts, complement levels (C3 and C4), C‐reactive protein, antinuclear antibody titers, and anti–double‐stranded DNA levels. Estimated glomerular filtration rate (eGFR) was calculated using the Chronic Kidney Disease Epidemiology Collaboration (CKD‐EPI) equation based on serum creatinine. Histopathologic variables included LN class, activity and chronicity indices, crescent presence and subtype, proportion of glomeruli affected by crescents, glomerulosclerosis, interstitial inflammation, tubular atrophy, interstitial fibrosis, immunofluorescence patterns, and EM findings. Treatment‐related variables included induction and maintenance immunosuppressive regimens and adjunctive therapies. Renal outcomes assessed during follow‐up included treatment response, changes in kidney function, reduction in proteinuria, requirement for renal replacement therapy, and a composite adverse renal outcome.

### 2.1. Statistical Analysis

Continuous variables are presented as mean ± standard deviation or median (interquartile range), and categorical variables as counts and percentages. Comparisons between crescentic and non‐crescentic groups were made using *t*‐tests or Mann–Whitney *U* tests for continuous variables, and Chi‐square or Fisher’s exact tests for categorical variables. Ordinal variables, such as LN class, activity and chronicity scores, and crescent subtype, were analyzed using Chi‐square tests for trend.

Event‐free survival for the composite renal outcome was estimated using Kaplan–Meier curves, and differences between groups were assessed with the log‐rank test. Cox proportional hazards regression was used to evaluate clinical and pathological factors associated with adverse renal outcomes. Variables with *p* < 0.1 on univariate analysis were included in multivariable Cox regression models, and hazard ratios (HR) with 95% confidence intervals (CI) are reported. Analyses were performed on all available data for each variable. Given the exploratory nature of the study and the predefined clinical and histopathological variables, no formal correction for multiple testing was applied. Results were interpreted with caution, taking into account clinical relevance and overall consistency of the findings in addition to statistical significance.

Descriptive and inferential analyses were conducted using SPSS version 30 (IBM Corp., Armonk, NY, United States), while advanced regression modeling and data visualization were performed in R Version 4.5.1 (R Foundation for Statistical Computing, Vienna, Austria). The manuscript text was reviewed and edited for clarity and readability only, with the assistance of ChatGPT (OpenAI, GPT‐4). All content was verified and approved by the authors.

## 3. Results

### 3.1. Baseline Characteristics

A total of 386 patients underwent renal biopsy. Hundred (100) patients with biopsy‐proven LN were included. Forty‐one out of 100 (41%) had crescents. None of the diabetics in our cohort has diabetic kidney disease. Baseline characteristics, histopathology, and treatment are summarized in Tables 1 and 2. Crescentic patients had significantly lower eGFR (71.41 ± 43.92 vs. 100.12 ± 45.63 mL/min/1.73 m^2^, *p* = 0.002) and serum albumin (25.51 ± 6.17 vs. 29.81 ± 7.56 g/L, *p* = 0.004) compared with non‐crescentic patients. Complement levels were also reduced in the crescent group (C3 0.58 ± 0.26 vs. 0.76 ± 0.44 g/L, *p* = 0.025; C4 0.12 ± 0.08 vs. 0.19 ± 0.14 g/L, *p* = 0.009), and ANA titers were higher (833.94 ± 501.91 vs. 500.00 ± 523.75, *p* = 0.008). Crescentic patients exhibited markedly higher activity scores (Modified NIH Activity Score 7.93 ± 3.05 vs. 2.27 ± 2.33, *p* < 0.001), while chronicity scores were similar between groups.

**Table 1:** Baseline clinical characteristics of lupus nephritis patients stratified by crescent.

Variable	Non‐crescent	Crescent	Total	*p* value
	59	41	100	
Age at diagnosis (years)	28.32 ± 14.97	27.20 ± 19.99	27.86 ± 13.12	0.675†
Systolic BP (mmHg)	125.77 ± 24.02	134.31 ± 32.71	129.24 ± 28.03	0.144†
Diastolic BP (mmHg)	74.49 ± 15.07	81.08 ± 18.90	77.17 ± 16.95	0.061†
Female	35 (85.4%)	49 (83.1%)	84 (84.0%)	0.76§
Male	6 (14.6%)	10 (16.9%)	16 (16.0%)	
Hypertension (yes)	30 (75.0%)	41 (69.5%)	71 (71.7%)	0.55§
Hypertension (no)	10 (25.0%)	18 (30.5%)	28 (28.3%)	
Diabetes (Yes)	8 (19.5%)	7 (12.5%)	15 (15.5%)	0.35§
Diabetes (No)	33 (80.5%)	49 (87.5%)	82 (84.5%)	
24‐h urine protein (g)	2.74 ± 3.86	3.80 ± 3.55	3.17 ± 3.75	0.166†
Serum creatinine (*μ*mol/L)	107.38 ± 138.19	148.52 ± 148.29	123.92 ± 143.02	0.166†
eGFR (mL/min/1.73 m^2^)	100.12 ± 45.63	71.41 ± 43.92	88.35 ± 46.91	**0.002** ∗ **†**
Serum albumin (g/L)	29.81 ± 7.56	25.51 ± 6.17	28.09 ± 7.32	**0.004** ∗ **†**
Hemoglobin (g/dL)	10.57 ± 2.03	10.19 ± 1.53	10.42 ± 1.85	0.312†
WBC count (×10^9^/L)	7.27 ± 3.82	6.90 ± 4.09	7.10 ± 3.93	0.694†
Platelet count (×10^9^/L)	222.44 ± 157.03	248.33 ± 122.25	233.53 ± 142.94	0.415†
C3 (g/L)	0.76 ± 0.44	0.58 ± 0.26	0.69 ± 0.39	**0.025** ∗ **†**
C4 (g/L)	0.19 ± 0.14	0.12 ± 0.08	0.16 ± 0.13	**0.009** ∗ **†**
CRP (mg/L)	8.29 ± 13.71	17.58 ± 32.92	11.88 ± 23.40	**0.062** ∗ **†**
ANA (index/titer unit)	1.16 ± 0.37	1.10 ± 0.31	1.14 ± 0.35	0.516†
ANA titer	500.00 ± 523.75	833.94 ± 501.91	655.21 ± 536.93	**0.008** ∗ **†**
Anti–dsDNA (index)	1.40 ± 0.62	1.20 ± 0.58	1.34 ± 0.61	0.162†
Anti–dsDNA titer	209.34 ± 556.51	115.86 ± 156.90	172.14 ± 443.78	0.31†
Anti‐Sm antibody (index)	14.30 ± 77.91	3.21 ± 11.36	9.15 ± 57.43	0.421
Duration to last follow‐up (y)	3.14 ± 3.09	3.24 ± 3.61	3.19 ± 3.29	0.882†

*Note:* Bold values indicate statistically significant differences (*p* < 0.05).

† Independent samples t‐test.

§ Chi‐square test.

¶ Fisher′s exact test.

**Table 2:** Histopathological and treatment characteristics of lupus nephritis patients according to crescent status.

Variable		Non‐crescent 59	Crescent 41	Total 100	*p* value
Austin NIH Activity Index		2.85 ± 3.42	7.39 ± 3.68	4.71 ± 4.17	**0.001** ^∗**†**^
Austin NIH Chronicity Index		2.37 ± 2.46	2.71 ± 1.93	2.51 ± 2.25	0.467v
Modified NIH Activity Score		2.27 ± 2.33	7.93 ± 3.05	4.59 ± 3.84	**< 0.001** ∗ **v**
Chronicity Score		2.64 ± 2.55	3.46 ± 2.19	2.98 ± 2.43	0.098†
Lupus class	Class I/II	2 (4.9%)	6 (10.2%)	8 (8.0%)	0.04
	Class III	5 (12.2%)	20 (33.9%)	25 (25.0%)	
	Class III and V	6 (14.6%)	7 (11.9%)	13 (13.0%)	
	Class IV	10 (24.4%)	9 (15.3%)	19 (19.0%)	
	Class IV and V	12 (29.3%)	6 (10.2%)	18 (18.0%)	
	Class V/VI	6 (14.6%)	11 (18.6%)	17 (17.0%)	
Activity/chronicity	A	4 (10.0%)	9 (16.4%)	13 (13.7%)	**< 0.001** ∗
	C	0 (0.0%)	19 (34.5%)	19 (20.0%)	
	AC	36 (90.0%)	27 (49.1%)	63 (66.3%)	
EM mesangial deposits	EM not done	12 (30.0%)	5 (8.9%)	17 (17.7%)	**< 0.001** ∗§
	Yes	10 (25.0%)	32 (57.1%)	42 (43.8%)	
	No	18 (45.0%)	19 (33.9%)	37 (38.5%)	
Podocyte effacement	≥50%	15 (37.5%)	34 (58.6%)	49 (50.0%)	0.07§
	<50%	9 (22.5%)	12 (20.7%)	21 (21.4%)	
	Not done	16 (40.0%)	12 (20.7%)	28 (28.6%)	
Induction drug	Cyclophosphamide	11 (26.8%)	13 (22.4%)	24 (24.2%)	0.61§
	MMF	30 (73.2%)	45 (77.6%)	75 (75.8%)	
Maintenance	MMF	38 (100.0%)	42 (76.4%)	80 (86.0%)	**0.01** ∗
	AZA	0 (0.0%)	6 (10.9%)	6 (6.5%)	
	Others	0 (0.0%)	7 (12.7%)	7 (7.5%)	
Full house	Full house	19 (52.8%)	32 (62.7%)	51 (58.6%)	0.35§
	Partial	17 (47.2%)	19 (37.3%)	36 (41.4%)	
Statin	Yes	16 (41.0%)	18 (31.0%)	34 (35.1%)	0.31§
	No	23 (59.0%)	40 (69.0%)	63 (64.9%)	
ACE inhibitor	Yes	22 (62.9%)	29 (58.0%)	51 (60.0%)	0.65§
	No	13 (37.1%)	21 (42.0%)	34 (40.0%)	
ARB	Yes	17 (44.7%)	28 (57.1%)	45 (51.7%)	0.25§
	No	21 (55.3%)	21 (42.9%)	42 (48.3%)	
SGLT‐2 inhibitor	Yes	3 (7.7%)	5 (8.5%)	8 (8.2%)	0.89
	No	36 (92.3%)	54 (91.5%)	90 (91.8%)	

Abbreviations: ACE, angiotensin‐converting enzyme; ANA, antinuclear antibody; ARB, angiotensin II receptor blocker; AZA, azathioprine; BP, blood pressure; CRP, C‐reactive protein; dsDNA, double‐stranded DNA; eGFR, estimated glomerular filtration rate; EM, electron microscopy; MMF, mycophenolate mofetil; NIH, National Institutes of Health; SGLT2, sodium‐glucose co‐transporter 2; WBC, white blood cell.

† †Independent sample *t*‐test.

^§^^§^Chi square test.

^¶^^¶^Fisher’s exact test.

Histopathologically, crescentic patients had a higher proportion of proliferative lupus classes (Class III 33.9% vs. 12.2%, Class III–V 11.9% vs. 14.6%, *p* = 0.04) and more active lesions (activity score C 34.5% vs. 0%, *p* < 0.001). EM detected mesangial deposits were more frequent in the crescent group (57.1% vs. 25.0%, *p* < 0.001), and there was a trend toward greater podocyte effacement (≥ 50% in 58.6% vs. 37.5%, *p* = 0.07).

Regarding renal outcomes, patients with ≥ 7.39% crescents had worse kidney function, with significantly higher rates of GFR < 20 mL/min/dialysis or doubling of serum creatinine (10/12 vs. 10/38 in the non‐crescent group, *p* = 0.045) and a higher proportion reaching the composite adverse outcome (12/12 vs. 15/36, *p* = 0.0460) [Table 3]. Complete and partial treatment response rates did not differ significantly by crescent burden (*p* = 0.294).

**Table 3:** Clinical and kidney outcomes according to the presence of active crescents and crescent burden.

Outcome category		Active crescent			Crescents			
		No	Yes	*p* value	None	< 7.39%	≥ 7.39%	*p* value
Composite outcomes (eGFR < 20 or doubling creatinine, dialysis, transplant)	Yes	14	14		15	1	12	
	No	37	25	0.391	36	12	14	0.046§
Response to treatment	Complete	26	11		25	6	6	
(Complete/partial/no response)	Partial	14	10		13	2	9	
	No	9	14	0.059	11	4	8	0.294§
Kidney function outcomes	EGFR < 20 mL per minute/dialysis	9	11		10	0	10	
	Doubling creatinine	3	2		3	0	2	
	Normal	39	26	0.49	38	13	14	0.045§
Reduction of proteinuria by 50%	Yes	17	20		17	11	9	
	No	18	9	0.1	19	3	5	0.113§

*Note:* Composite outcome defined as eGFR <20 mL/min/1.73 m^2^, doubling of serum creatinine, dialysis, or kidney transplantation. Data are presented as the number of patients. Percentages were calculated based on available data. Abbreviations: eGFR, estimated glomerular filtration rate.

§Chi‐square test.

### 3.2. Clinical and Pathological Characteristics Stratified by Proteinuria

**Figure 1:**
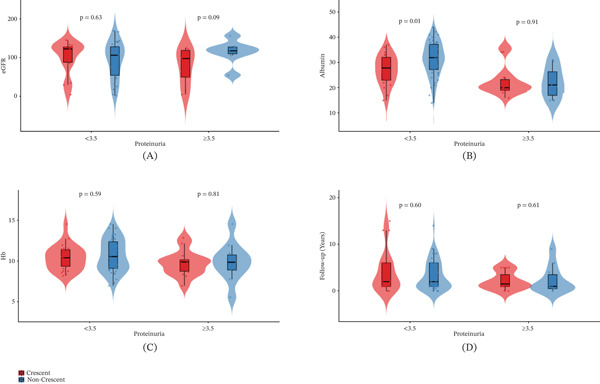
The figure shows violin plots comparing clinical parameters between crescentic and non‐crescentic lupus nephritis stratified by proteinuria (< 3.5 vs ≥ 3.5 g/day).

Figure 1 shows the distribution of key clinical and histopathologic parameters in patients with crescentic and non‐crescentic LN. These parameters were stratified by 24‐h proteinuria (< 3.5 g/day vs. ≥ 3.5 g/day). Among patients with high proteinuria (≥ 3.5 g/day), eGFR tended to be lower in those with crescents than in those without crescents; however, this difference did not reach statistical significance (*p* = 0.09). On the other hand, in patients with low proteinuria (< 3.5 g/day), eGFR did not differ between groups. Serum albumin was significantly lower in crescentic patients with proteinuria < 3.5 g/day, whereas no difference was observed in those with proteinuria ≥ 3.5 g/day. Hemoglobin levels and follow‐up duration were similar between groups across both proteinuria strata (all *p* > 0.05).

**Figure 2:**
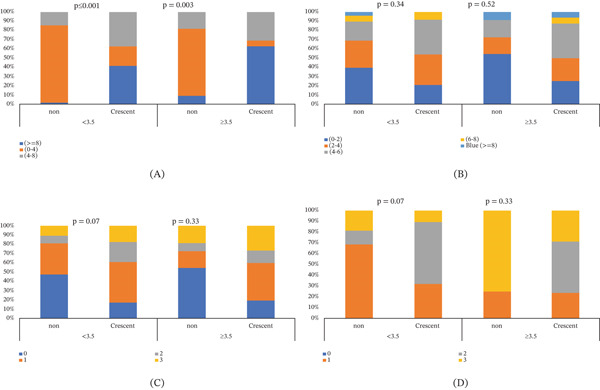
Stacked bar charts comparing (A) histologic activity score, (B) chronicity score, (C) endocapillary hypercellularity, and (D) neutrophil/karyorrhexis between crescentic and non‐crescentic lupus nephritis, stratified by proteinuria level.

Stacked bar charts (Figure 2) comparing histologic activity (AI score), chronicity (CI score), endocapillary hypercellularity, and neutrophil/karyorrhexis demonstrated that crescentic cases had a higher proportion of high AI scores (≥ 8) in both low and high proteinuria groups (*p* ≤ 0.001 and *p* = 0.003, respectively). CI score distributions were comparable between crescentic and non‐crescentic cases regardless of proteinuria level. Endocapillary hypercellularity and neutrophil/karyorrhexis tended to be more pronounced in crescentic nephritis with low proteinuria, but differences were not statistically significant in the high proteinuria group.

### 3.3. Clinical and Histopathologic Predictors of Adverse Renal Outcomes by Cox Regression

Cox regression analyses were performed to identify clinical and pathological factors associated with the composite renal outcome. In patients with crescents, older age at diagnosis, higher baseline serum creatinine, lower complement C3 levels, and elevated CRP were associated with increased risk of adverse outcomes (Table 4). Among the pathological features, fibrous crescents were significantly associated with a higher hazard. Other lesions, such as glomerulosclerosis, interstitial fibrosis, tubular atrophy, and interstitial inflammation, were not significant (Table 4).

**Table 4:** Cox regression for (A) clinical and (B) pathological risk factors associated with adverse composite outcome in lupus nephritis with crescents and proliferative lupus nephritis.

Variables	Crescent (*n* = 41)		Proliferative nephritis (*n* = 75)	
	HR [95% CI]	*p* value	HR [95% CI]	*p* value
A—Clinical risk factors				
Age at time of diagnosis	1.13 [1.02–1.25]	**0.02** ∗	1.07 [1.01–1.13]	**0.015** ∗
Gender	2.13 [0.51–8.95]	0.302	4.20 [1.22–14.49]	**0.023** ∗
24‐h urine protein (g)	0.79 [0.59–1.06]	0.11	1.00 [0.80–1.24]	0.965
Serum creatinine at baseline	1.00 [1.00–1.01]	**0.014** ∗	1.00 [1.00–1.01]	**0.001** ∗
ANA titer	1.00 [1.00–1.00]	0.532	1.00 [1.00–1.00]	0.6
C3	0.05 [0.00–0.94]	**0.045** ∗	0.87 [0.13–5.83]	0.887
CRP	1.03 [1.01–1.06]	**0.005** ∗	1.03 [1.01–1.04]	**0.003** ∗
B—Pathological risk factors				
Total glomerulosclerosis (global + segmental)	0.40 [0.15–1.08]	0.071	1.30 [0.78–2.15]	0.318
Fibrous crescents	4.52 [1.11–18.30]	**0.035** ∗	0.99 [0.53–1.84]	0.964
Interstitial fibrosis	0.66 [0.06–7.83]	0.743	1.91 [0.86–4.27]	0.112
Tubular atrophy	0.66 [0.06–7.83]	0.743	1.65 [0.36–7.47]	0.518
Interstitial inflammation	2.13 [0.86–5.30]	0.104	0.70 [0.16–3.14]	0.641
Neutrophil/karyorrhexis	1.19 [0.63–2.24]	0.595	2.10 [1.34–3.28]	**0.001** ∗
Fibrinoid necrosis ×2	1.04 [0.60–1.80]	0.885	1.26 [0.97–1.65]	0.087

*Note:* Bold values indicate statistically significant results (*p* < 0.05). An asterisk (∗) denotes statistical significance in the multivariable analysis.

Abbreviations: ANA, antinuclear antibody; CI, confidence interval; CRP, C‐reactive protein; HR, hazard ratio.

In the overall LN cohort, the presence of any crescents, particularly ≥ 7.39% crescent burden, was associated with a higher risk of reaching the composite outcome in univariate analysis (Table 5). After adjustment for other clinical and pathological variables, these associations were attenuated and no longer statistically significant. Similarly, in patients with proliferative LN, univariate analysis suggested a higher risk with any crescents or high crescent burden, but these associations were not significant in multivariable models (Table 6).

**Table 5:** Cox regression analysis: (A) Correlations between the presence of crescents and composite outcomes in all patients with lupus nephritis; (B) correlations between 7.39% of glomeruli with crescents and composite outcomes in all patients with lupus nephritis.

Variable	Univariate		Multivariable	
	HR [95% CI]	*p* value	HR [95% CI]	*p* value
A—Crescents				
No	1		1	
Yes	3.60 [1.19–10.89]	**0.024** ∗	3.10 [0.56–17.16]	0.196
B—Crescents				
No	1		1	0.256
< 7.39%	0.63 [0.08–5.38]	0.676	0.28 [0.02–3.19]	0.303
≥ 7.39%	3.58 [1.38–9.27]	**0.009** ∗	1.57 [0.35–7.10]	0.558

*Note:* Bold values indicate statistically significant results (*p* < 0.05). An asterisk (∗) denotes statistical significance in the multivariable analysis.

Abbreviations: CI, confidence interval; HR, hazard ratio.

**Table 6:** Cox regression analysis: (A) Correlations between the presence of crescents and composite outcomes in patients with proliferative lupus nephritis; (B) correlations between 7.39% of glomeruli with crescents and composite outcomes in patients with proliferative lupus nephritis.

Variable	Univariate		Multivariable	
	HR [95% CI]	*p* value	HR [95% CI]	*p* value
**A—Crescents**				
**No**	1		1	
**Yes**	3.95 [1.05–14.93]	**0.043** ^∗^	4.57 [0.61–34.39]	0.14
**B—Crescents %**				
**No**	1		1	0.256
**< 7.39%**	1.33 [0.13–14.17]	0.813	0.52 [0.04–7.08]	0.62
**≥ 7.39%**	2.95 [0.88–9.87]	0.079	1.16 [0.23–5.95]	0.857

*Note:* *p* < 0.05 is considered significant.

Abbreviations: CI, confidence interval; HR, hazard ratio.

^∗^Reference categories: No crescents or < 7.39% crescents.

### 3.4. Event‐Free Survival According To Crescent Presence and Burden

**Figure 3:**
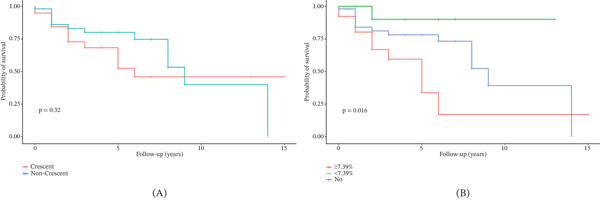
(A) Kaplan–Meier survival analysis comparing patients with and without active crescents. Survival distributions did not differ significantly between groups (log‐rank [Mantel–Cox] test, *χ* ^2^ = 0.97, df = 1, *p* = 0.324). (B) Kaplan–Meier survival curves comparing outcomes across crescent severity groups stratified using a cutoff of 7.39%. Survival distributions differed significantly between groups (log‐rank [Mantel–Cox] test, *χ* ^2^ = 8.25, df = 2, *p* = 0.016).

The Kaplan–Meier method was used to estimate event‐free survival for the composite renal outcome. Overall, there was no statistically significant difference between patients with and without crescents (*p* = 0.32), although the survival curve for crescentic patients tended to be lower throughout follow‐up. When patients were stratified by crescent burden, those with ≥ 7.39% of glomeruli affected had the poorest event‐free survival, suggesting a higher risk of adverse outcomes. Patients with < 7.39% crescents showed the most favorable prognosis, while patients without crescents had intermediate survival (Figure 3). This indicates that the extent of crescents, rather than their mere presence, may have a greater influence on renal outcomes.

## 4. Discussion

This is the first study conducted on crescents and their prognostic value from Saudi Arabia. We found a couple of pertinent findings. In our cohort, crescent formation was frequently observed in patients with LN and was associated with a more active disease phenotype at presentation. Patients with crescents had worse renal function, lower serum albumin, greater complement consumption, and higher autoantibody levels. Histologically, crescents were mainly observed in proliferative LN and were associated with higher activity scores. Chronicity scores were comparable between crescentic and non‐crescentic disease. Although patients with a higher crescent burden had poorer renal outcomes during follow‐up, crescent burden alone was not sufficient to predict prognosis. Notably, treatment response rates were similar across crescent burdens, indicating that short‐term therapeutic response may not fully capture the long‐term prognostic impact of extensive crescent formation. Because treatment regimens were not standardized, these results should be interpreted with caution.

Despite the relatively high burden of LN in Saudi Arabia, published data remain limited. Most studies are retrospective, single‐center, and show wide variability in reported outcomes, with little attention to specific prognostic histopathologic features such as crescents [8]. Similar patterns are seen across the Middle East, where reports are largely descriptive and heterogeneous [9]. Overall, the region appears to have a higher proportion of proliferative disease, but detailed evaluation of histopathologic prognostic markers is still lacking, underscoring the need for larger multicenter studies [10].

Baseline characteristics showed that patients with crescent formation had more active disease at presentation. These patients had significantly lower eGFR and serum albumin levels, along with greater complement consumption and more severe renal and immunologic involvement. Similar findings have been reported by Lu et al., who showed that patients with crescentic LN presented with worse renal function and more active immunologic disease at diagnosis [15]. Crescent formation was mainly observed in proliferative LN and was associated with higher NIH and modified activity scores, whereas chronicity scores were similar between groups. Consistent with our results, Lin et al. found that crescent formation in LN was predominantly associated with proliferative histologic classes and higher renal activity indices, supporting the interpretation that crescents primarily indicate active inflammatory injury rather than chronic damage [16]. Together, these observations support the interpretation that crescents are a marker of active inflammatory injury in LN.

Examining patients by proteinuria level revealed several patterns. Although patients with high proteinuria and crescents showed a trend toward lower eGFR compared with those without crescents, this difference did not reach statistical significance. Whereas this difference was not seen in patients with lower proteinuria. In the low‐proteinuria group, serum albumin was lower in patients with crescentic glomerulonephritis. Histologically, crescentic cases had higher activity scores across both proteinuria groups, while chronicity scores were similar between groups. Features of active glomerular inflammation, including endocapillary hypercellularity and neutrophil/karyorrhexis, were more evident in crescentic cases with lower proteinuria, whereas these differences were less marked in the high‐proteinuria group. Although prior studies have shown that the prognostic impact of crescents is attenuated in patients with nephrotic‐range proteinuria [17], this does not imply a favorable prognosis. Rather, nephrotic‐range proteinuria itself identifies a uniformly high‐risk group, in whom the presence of additional histologic features, such as crescents, may provide only limited incremental prognostic discrimination. In our cohort, patients with nephrotic‐range proteinuria had poor outcomes, in keeping with this concept. Importantly, among patients with lower proteinuria, hypoalbuminemia identified a subgroup with worse outcomes; this may reflect that serum albumin reflects disease activity and systemic inflammation beyond proteinuria level alone. These subgroup analyses are exploratory and should be interpreted cautiously, given the small sample sizes. Overall, these findings indicate that crescents, proteinuria, and serum albumin provide complementary information about disease severity.

In our study, several clinical and pathological factors were linked to worse outcomes in LN. Clinically, older age at diagnosis, higher baseline serum creatinine, and elevated CRP were associated with a higher risk in both crescentic and proliferative disease. Although baseline gender distribution was similar between groups, females were predominant in the overall cohort, and male sex emerged as an independent predictor after adjustment, highlighting a potential sex‐based difference in disease severity. Various studies showed that older age [18, 19], higher baseline creatinine [17, 18] and elevated C‐reactive protein were associated with worse renal outcomes [18]. Low C3 levels were associated with poor outcomes in patients with crescents, but neither proteinuria nor ANA titer independently predicted the composite endpoint. Other studies have also implicated C3 hypocomplementemia in worse renal outcomes in LN [19, 20]. On the pathological level, fibrous crescents were consistently associated with adverse outcomes in crescentic LN. Supporting this, Rao et al. reported that patients with fibrous crescents had the poorest prognosis, with higher rates of treatment resistance and mortality compared with those with cellular or fibrocellular crescents [21]. On the other hand, neutrophil/karyorrhexis stood out as a risk factor for proliferative disease. Similar to our findings, Magil et al. [22] demonstrated that in diffuse proliferative LN, the extent of karyorrhexis, reflecting active neutrophil‐mediated injury, was a significant prognostic factor and was associated with worse renal outcomes independent of other histologic features.

While investigating the prognostic effects of crescents, we observed several important observations. In the overall LN cohort, crescent presence and higher crescent burden (≥ 7.39% of glomeruli) were associated with adverse outcomes in univariate analysis, but these associations lost significance after multivariable adjustment. In contrast, when individual crescent subtypes were examined, the presence of fibrous crescents remained significantly associated with a higher risk, suggesting that crescent subtype may be more relevant than crescent quantity alone for long‐term prognosis. A lower crescent burden (< 7.39%) was not associated with worse outcomes, which might suggest that limited crescent involvement may not confer excess risk. Kaplan–Meier analysis showed that patients with ≥ 7.39% crescents had the poorest event‐free survival, those with < 7.39% crescents had the best event‐free survival, and patients without crescents had intermediate outcomes; however, these differences likely reflect baseline disease severity rather than independent prognostic effects. Taken together, these findings suggest that while crescent burden reflects disease severity, it does not independently predict adverse renal outcomes. In contrast, Tao et al. [13] reported higher crescent burden as an independent predictor in their cohort, a difference that may relate to variations in baseline renal function, histologic activity and chronicity, treatment strategies, and follow‐up duration. In our analysis, increasing age, baseline creatinine, elevated C‐reactive protein, and the presence of fibrous crescents were associated with renal outcomes. Our findings are in line with those of Moroni et al. [23], who reported that crescents were not independent predictors of long‐term renal outcomes, whereas clinical factors such as nephritic flares and failure to achieve complete remission were more predictive. Similarly, another study showed that although glomerular crescents were associated with poorer outcomes on univariate analysis, tubulointerstitial lesions, tubular atrophy, and baseline renal dysfunction were stronger independent predictors of long‐term renal survival, supporting the view that the prognostic impact of crescents is largely influenced by accompanying non‐glomerular injury [24]. Overall, these data suggest that crescents are better viewed as markers of disease severity and inflammatory activity rather than standalone predictors of long‐term prognosis.

This study has several strengths. It included patients from two major centers in Saudi Arabia, allowing inclusion of a broader spectrum of clinical practice than single‐center studies. Only biopsy‐proven LN cases with complete clinical, lab, and pathology data were included, and all biopsies were independently reviewed by two pathologists using the updated ISN/RPS 2018 criteria, ensuring reliable histologic assessment. We looked not only at the presence of crescents but also at their type and burden, and analyzed outcomes using well‐defined composite endpoints and multivariable models. However, there are limitations. The study is retrospective, so causality cannot be established, and treatment regimens were not standardized, which may have influenced outcomes. The sample size, particularly for patients with high crescent burden, was relatively small. Follow‐up times varied, and repeat biopsies were unavailable, while the cutoff for high crescent burden was taken from previous studies and may not apply to all populations.

## 5. Conclusion

In conclusion, crescents in LN were associated with more active disease, lower kidney function, and greater immunologic activity, especially in proliferative cases. Although patients with a higher crescent burden had worse outcomes in univariate analysis, crescents were not independent predictors after adjusting for other clinical and pathological factors. This suggests that crescents mainly reflect disease activity rather than long‐term prognosis on their own. Considering crescent presence and extent, along with other clinical and laboratory markers, can help identify patients at higher risk and guide management. These findings provide important insight into the role of crescents in Saudi patients with LN.

**Nomenclature**
AI: activity index
ANA: antinuclear antibody
anti‐dsDNA: anti–double‐stranded deoxyribonucleic acid antibody
C3: complement component 3
C4: complement component 4
CI: chronicity index
CI (statistical): confidence interval
CRP: C‐reactive protein
eGFR: estimated glomerular filtration rate
EM: electron microscopy
GFR: glomerular filtration rate
HE: hematoxylin and eosin
HR: hazard ratio
IBM: international business machines
ISN/RPS: International Society of Nephrology/Renal Pathology Society
LN: lupus nephritis
NIH: National Institutes of Health
PAS: periodic acid–schiff
SPSS: Statistical Package for the Social Sciences
WBC: white blood cell

## Author Contributions

S.H.A.‐Q., M.A.M.K., and N.M.S.: conceptualization. H.H.K.M., F.A.A., A.M.A., R.M.H.A., L.A., M.M.A., and R.A.A.: data curation. A.E.: formal analysis. S.H.A.‐Q., M.A.M.K., and N.M.S.: investigation. M.A.M.K. and S.H.A.‐Q.: methodology. S.H.A.‐Q. and N.M.S.: supervision. A.E.: visualization. M.A.M.K.: writing — original draft. S.H.A.‐Q., M.A.M.K., H.H.K.M., F.A.A., A.M.A., R.M.H.A., L.S.A., M.M.A., R.A.A., A.E., and N.M.S.: writing — review and editing.

## Funding

No funding was received for this manuscript.

## Disclosure

No financial support or sponsorship influenced any aspect of the study.

## Ethics Statement

The study protocol was approved by the Research Ethics Committee of King Fahad Armed Forces Hospital, Jeddah. All procedures were conducted in accordance with the Declaration of Helsinki. This retrospective observational study was conducted and reported in accordance with the Strengthening the Reporting of Observational Studies in Epidemiology (STROBE) guidelines.

## Conflicts of Interest

The authors declare no conflicts of interest.

## Data Availability

The data that support the findings of this study are available on request from the corresponding author. The data are not publicly available due to privacy or ethical restrictions.
